# Foreign investment in emerging legal medicinal cannabis markets: the Jamaica case study

**DOI:** 10.1186/s12992-021-00687-3

**Published:** 2021-04-01

**Authors:** Marta Rychert, Machel Anthony Emanuel, Chris Wilkins

**Affiliations:** 1 Shore & Whāriki Research Centre, College of Health, Massey University, Auckland, New Zealand; 2 Department of Life Sciences, University of the West Indies, Mona Campus, Kingston, Jamaica

**Keywords:** Cannabis, Policy, Legalisation, Medical cannabis, Cannabis industry, Caribbean, Jamaica

## Abstract

**Introduction:**

The establishment of a legal market for medicinal cannabis under the Dangerous Drugs Amendment Act 2015 has positioned Jamaica at the forefront of cannabis law reform in the developing world. Many local cannabis businesses have attracted investment from overseas, including from Canada, US and Europe.

**Aim:**

To explore the opportunities and risks of foreign investment in an emerging domestic legal cannabis market in a developing country.

**Methods:**

Thematic analysis of semi-structured face-to-face interviews with 22 key informants (KIs) from the Jamaican government, local cannabis industry, academia and civil society, and field observations of legal and illegal cannabis cultivators.

**Results:**

KIs from the Jamaican public agencies and domestic cannabis entrepreneurs saw foreign investment as an essential source of capital to finance the start-up costs of legal cannabis businesses. Local cannabis entrepreneurs prioritised investors with the greatest financial resources, brand reputation and export networks. They also considered how allied an investor was with their business vision (e.g., organic cultivation, medical vs. recreational). The key benefits of partnering with a foreign investor included transfer of technical knowledge and financial capital, which enhanced production, quality assurance and seed-to-sale tracking. Some KIs expressed concern over investors’ focus on increasing production efficiency and scale at the expense of funding research and development (R&D) and clinical trials. KIs from the local industry, government agencies and civil society highlighted the risks of ‘predatory’ shareholder agreements and domestic political interference. Concerns were raised about the impact of foreign investment on the diversity of the domestic cannabis sector in Jamaica, including the commitment to transition traditional illegal small-scale cannabis cultivators to the legal sector.

**Conclusion:**

While foreign investment has facilitated the commercialisation of the cannabis sector in Jamaica, regulatory measures are also needed to protect the domestic industry and support the transition of small-scale illegal cultivators to the legal regime. Foreign investments may alter the economic, social and political determinants of health in transitioning from illegal to legal cannabis market economy.

## Introduction

In the past decade, 15 US states, Uruguay and Canada have legalised markets for recreational cannabis and many more jurisdictions have liberalised their medicinal cannabis laws [28, 38]. With increasing policymaker interest in cannabis law reforms around the world, the potential for international trade in cannabis is growing. Some of the biggest cannabis companies in the ‘Global North’ have established operations across continents [43]. For example, Canadian-based Canopy Growth™ holds licenses for cultivation and production in Europe (Denmark) and South America (Colombia) (having recently exited operations in South Africa and Lesotho), with access to more cannabis markets via joint ventures and partnerships across the world [10, 11]. Other Canadian-based cannabis companies have adopted a similar global trade model: Cronos Group™ operates across five continents (including joint ventures in Israel, Colombia and Australia), Aurora™ is present in 25 countries; and Aphria™ (which announced a merger with Tilray™ in 2020) operates in at least ten countries [3, 4, 18].

The constraints of the United Nations international drug control treaties and the relatively small number of jurisdictions where recreational cannabis use is legal currently limit the extent of the global trade in cannabis to medicinal and scientific sectors only. However, the interrelationship between the medicinal and recreational cannabis sectors is becoming increasingly clear [27] and some cannabis firms in legal jurisdictions now trade in both medicinal and recreational cannabis markets.

Many commentators have warned against the dangers of “Big Cannabis”, the corporatised industry model known from the alcohol, tobacco and pharmaceutical sector [7, 40]. Alcohol and tobacco researchers have highlighted problems with the global industry model, including the financial power of international corporations to lobby governments and challenge public health measures via international trade courts; concerted efforts to distort scientific evidence about harms; and the use of similar corporate social responsibility strategies across countries and continents [5, 51]. Developing economies are particularly vulnerable to these political and economic pressures, and international alcohol and tobacco corporations have replaced local, unsophisticated businesses coming in with promises of economic growth, including foreign direct investment and employment opportunities [22, 24, 25, 39, 42].

Information regarding the strategies and operations of international investors in the emerging legal cannabis markets is currently limited. Investors in the ‘Global South’ (i.e., Africa, Latin America and South-East Asia) can potentially benefit from favourable climate conditions and lower labour costs. A number of developing countries have recently reformed their drug laws and established legal medicinal cannabis regimes in the hope of attracting international investment and boosting their economies, with a recent reform in Malawi – one of the poorest countries in the world, which followed a number of other African jurisdictions (Zimbabwe, Zambia, Lesotho and South Africa) [48]. Some commentators in Africa and South America have identified “strong colonial overtones” [8, 20] and warned against the “takeover of the [cannabis] market by large corporations” [47]. Yet, foreign investment in emerging cannabis markets can also provide much needed capital for research and infrastructural development, as evidenced in positive examples from other sectors [1, 41, 59]. This opportunity for investment and knowledge transfer may be particularly attractive in the *medicinal* cannabis sector, where production of cannabis for export needs to comply with standards for the international trade.

In April 2015, with the passage of the Dangerous Drugs Amendment Act 2015, Jamaica became the first country in the Caribbean to establish a new legal regime for a domestic medicinal, therapeutic and scientific cannabis industry, thus positioning itself at the forefront of international cannabis reforms in the developing world. This research aims to explore the opportunities and threats of foreign investment in the emerging medicinal cannabis market in Jamaica. Specifically, our research objectives were to (1) understand *why* and *how* local stakeholders in Jamaica engaged with foreign investors, and (2) investigate how local cannabis policy stakeholders (i.e., local industry, policymakers, regulators and civil servants, academics and civil society) viewed the risks and opportunities that foreign investment has brought in the early stage of implementing the reform. The paper explores the role of international investors in the transition from illegal to legal medicinal cannabis market in Jamaica and their potential influence on the social, political and economic determinants of health. Better understanding of experiences in Jamaica may provide lessons for other developing countries that are considering similar cannabis reforms.

## Background: medicinal cannabis market and regulations in Jamaica

The establishment of a legal market for medicinal cannabis in Jamaica via the passage of the Dangerous Drugs Amendment Act (DDAA) 2015 was lauded as a significant economic opportunity for Jamaica’s developing economy, with the potential to contribute export earnings, attract foreign investment and encourage cannabis tourism [19, 33]. Under the DDAA, a new government agency (Cannabis Licensing Authority, CLA) was established with a mandate of developing detailed regulations for the new industry, issuing licenses and monitoring the compliance of licensed operators with regulatory requirements. The DDAA does not restrict the medical conditions that qualify for a doctor’s recommendation to legally obtain medicinal cannabis or the types of products that can be sold via CLA-licensed outlets (i.e. the regime accommodates both cannabis herb for smoking and processed products such as oils and extracts^1^) [54].

Jamaica has a significant cultural history of cannabis use and illegal cannabis cultivation, with an estimated 15,000 ha of cannabis cultivated annually illegally, mostly for export [61]. Sectors of Jamaican society have also traditionally used cannabis as a folk medicine and in religious ceremonies [13, 29]. The new legal medical cannabis regime was envisioned to increase legal employment for disadvantaged communities and the transitioning of illegal subsistence cannabis farmers to the new legal regime [26].

The implementation of the legal medicinal cannabis market has proved challenging, with long time frames for finalising regulatory frameworks (e.g., production standards and export regulations) and concerns over structural and financial barriers faced by small-scale illegal cannabis cultivators in transitioning to the new regime [34, 54, 60]. Local stakeholders have described the security and surveillance requirements for legal cannabis cultivation as “difficult and onerous”, reflecting the lawmakers’ focus on preventing diversion and ensuring compliance with international drug control framework [54]. The cost of security and surveillance infrastructure has been identified as one of the main barriers preventing the transition of traditional illicit cannabis farmers into the new regime, and for the domestic sector’s reliance on investors from overseas [37, 54].

Despite the challenges, as of August 2020, the CLA had granted 67 cannabis licenses across five license categories (i.e., cultivation, processing, transport, retail and research) [45]. Media reports have highlighted the presence of overseas companies investing in the local cannabis sector in Jamaica, including some of the largest Canadian cannabis firms [44, 53, 58]. However, there are no official figures on the extent of international investment in the Jamaican cannabis market as such data is considered confidential business information (insights can be gleaned from reports of individual investor companies listed on stock exchange, which were consulted as part of formative research for this study). As of August 2020, the majority of licenses for cultivation sites (60%) were granted for farms larger than 1 acre (i.e., Tier 2 and Tier 3 cultivation licenses), suggesting interest in large scale production. There is also evidence of vertical integration in the market, with a number of local licensed medicinal cannabis businesses marketing themselves as fully vertically integrated (e.g. [21, 31, 32, 35]).

Regulations under the DDAA include measures to protect local ownership of the medicinal cannabis industry, i.e.: licensed companies must demonstrate “substantial ownership and control by persons ordinarily resident in Jamaica”. This clause has been interpreted as a requirement for “over 50% of the shareholding and the directorship” to be held in Jamaican hands, with the exception of research and development (R&D) licences, where only 25% domestic ownership is required, reflecting the capital-intensive nature of research [54]. Domestic owners must also be “ordinarily residents” of Jamaica for the past 3 years.

## Method

We use the qualitative key informant interview method [12, 46] to explore stakeholders’ views and experiences with foreign investment in the emerging cannabis sector in Jamaica. A detailed description of the study methodology was provided in an earlier publication [54]. A concise summary of the method is provided below.

We conducted 22 in-depth face-to-face one-on-one interviews with key cannabis policy stakeholders in Jamaica. The key informants (KI) included: civil servants from health, business and enforcement agencies (7), licensed cannabis industry players including cultivators, processors and retailers (6), academics from the health, pharmaceutical and business fields (5), cannabis and social justice activists (2) and politicians (2). A semi-structured interview schedule was used to explore KIs’ experiences with the implementation of the cannabis law reforms, including foreign investment in the domestic cannabis sector. The interview schedule consisted of general questions asked of all KIs (e.g., views on the “majority ownership” rule, perceived benefits and disadvantages of foreign investment), and specific questions addressed to particular KI types (e.g., criteria used by domestic medicinal cannabis entrepreneurs looking for foreign investors). The interviews were conducted in November and December 2019, i.e., 4.5 years after the DDAA was passed in the Parliament. The average interview duration was 72 min. Interviews were recorded, transcribed and analysed thematically [9], with primary analytical focus on participants’ narratives about the economic, political, health and social benefits and disadvantages that arise from the presence of foreign investors in the domestic medicinal cannabis market in Jamaica. Field visits to licensed operators and illegal cannabis cultivators provided further context for the analysis. In line with the qualitative description approach [52, 56], rich descriptions of KIs’ experiences with foreign investors and views on investors’ role in the regime are presented in the results section. The study’s ethical protocols were registered with the Massey University Human Subjects Ethics Committee (ref. 19/37).

## Results

### Motivations for partnering with foreign investors

All local cannabis industry KIs (i.e., 6 businesses cumulatively holding 15 licenses at the time of interviewing) reported partnerships with overseas cannabis firms and pharmaceutical companies, primarily from Canada, but also from Europe and the US. Local industry KIs and public official KIs saw foreign direct investment as an essential source of capital to finance the start-up costs of medicinal cannabis businesses. Some industry KIs described unsuccessful efforts to attract domestic investors (e.g., KIs 16, 18, 19, industry) therefore illustrating some preference for local investment. Securing domestic partnerships, however, proved challenging due to Jamaican banks’ refusal to service the cannabis sector (in order to protect banking relationships with banks in the U.S., where cannabis remains prohibited at the federal level). Consequently, Jamaican cannabis entrepreneurs expressed a sentiment that they had ‘no choice’ but to partner with overseas companies in order to secure the necessary capital to meet the requirements of security and surveillance infrastructure:

I had to partner with [country], right? I didn’t have the money for myself. And I decided I am not going to be left out of this industry so if it means that I am going to partner with some white Canadian or some White Americans, I am going to make sure that I have a foot in the niche. (KI10, industry).

We have held in this very space three different investor sessions with people in Jamaica who *have* money. (…) You try to get an investor locally when they’re risking their solvency, when they’re risking their ability to trade, that’s not gonna to happen... (KI19, industry).

### Criteria for choosing international investors

While the extent of financial investment was key in deciding what foreign company to partner with, consistency between the foreign investor’s mission and the domestic entrepreneur’s vision was also considered important. For example, the quotes below illustrate how some domestic cannabis entrepreneurs expressed a preference for investors with a track record of organic cultivation and dedication to the medical segment of the cannabis market:

It’s a business and the purpose of a business is to make profit, but there also must be a commitment to the development of medicine for the humanity. And I am convinced that we have a mix of both. I have been approached by partners whose only purpose is to maximise profit and I have walked away. (KI8, industry).

[Company name] was one that stood out, because of the fact that they were the only organic company pushing for the organic side of things and we were on that same path as well. That was one of the major things that stood out, that was an attraction for us. (KI15, industry).

The benefits of partnering with foreign investors extended beyond solely monetary support and included factors such as the investor’s brand reputation and the size of potential export market, where Jamaican suppliers hoped to take advantage of the distribution systems already established by foreign investors. As illustrated in the quote below, this reflects local industry KIs’ perception that large cannabis players, with an internationally recognised brand and reputation, will provide the cost advantages necessary to remain competitive in the emerging global cannabis sector:

They [investors’ trade name] were doing more capital raises so it was all over the news that you know [investor’s name] raises x amount of hundreds of millions, so it was very much promoted, their name was promoted in the media so it was an easy sell, you know, to any stakeholder here. (…) it was in [year] I got to really test [investor’s name] products but before that it was just that they were one of the most cash-rich cannabis companies in the world. (KI14, industry).

### The benefits and gaps in the knowledge spillover and technology transfer

Asked about other benefits of overseas investment, civil servants and domestic industry KIs cited transfer of technological and market knowledge. Foreign investors have provided Jamaican entrepreneurs with access to cultivation technologies and business knowledge that increased the efficiency of cannabis sector in Jamaica. Key knowledge and technology transfer included developing the scalability of cannabis production and the implementation of efficient seed-to-sale monitoring and cannabis processing systems (e.g., extraction technologies and drying techniques). Domestic cannabis entrepreneurs stressed the positive impact of this technology transfer on the quality of their cannabis products:

Had we not had foreign partners we would still be using a smaller extraction system, [but] owning it 100%. And we would be patting ourselves on the back ‘oh, we own this 100%’. We would be probably able to process 10 pounds a day [now can process 100–150 pounds a day] and the quality … right? We have taken [cannabis] oil for experiment from other people who say they have good oil, and when we finished processing it, we have removed a lot of carbon, chlorophyll and made a refined product. (KI9, industry).

I will admit especially the drying techniques that we’ve learned [from them] - are very, very important to having better quality flowers. (KI14, industry).

Despite these reported technological and knowledge transfers, many KIs from academia and the government sector expressed concern over the limited investment in research and development (R&D), which would contribute to the development of value-added medicinal cannabis products. KIs referred to the “limited domestic [research] capabilities” (KI22, politician) such as laboratories and research equipment, where they thought more investment should be directed in the early stage of the regime, as argued in the following quote:

What you want is more strategic investment. You want business that is going to do the R&D, do the research, do the clinical trials, that is also going to help other sectors like medical tourism, wellness tourism, things like that. That is what you want. And then you want investment that is going to give you the ability to do value-added product. So you are not just growing the plant, you are manufacturing *medicines* locally. You want some of that strategic investment as well. So far that is a little bit disappointing. (KI5, civil servant).

### Perceived impact of foreign investment on public health

Two polar perspectives on the potential health impacts of foreign investment in the sector featured in the interviews. On one hand, many KIs from across private and public sectors stressed investors’ contribution to facilitating access to quality-assured cannabis products for therapeutic use. KIs with this view positioned the industry within the pharmaceutical and medical paradigm with medical patients and consumers being the primary beneficiaries of the reform (e.g. KI11, academic; KI18, industry). On the other hand, a couple of KIs with professional background in addiction and public health, considered that the presence of foreign investors exacerbates the challenges to the DDAA posed by the commercialisation of the medicinal cannabis sector (e.g. the extent of direct-to-consumer advertising, which is currently not regulated under the DDAA framework). For example, one KI said that “by having all of this international investment coming in, you are fuelling the expansion of the amendment that is already poorly implemented” (KI-1), reflecting on issues such as the marketing, insufficient medical oversight and the wider threat of the regime transitioning to “recreational” market model.

### Risks of “predatory” shareholder agreements

Despite the “substantial ownership” rule requiring that at least 50% of the shareholding and directorship needs to be held by domestic operators, some local entrepreneurs and civil servant KIs observed that international investors are able to exert influence over local companies, for example via strategic structuring of shareholder agreements. Local cannabis entrepreneurs gave examples of what they considered ‘predatory’ practices, e.g.: international investors obliging local businesses to not raise capital outside of the foreign partnership, or applying pressure to adopt cultivation and processing practices that local KIs considered not conducive to the Jamaican context. The latter included investors insisting on cultivating foreign cannabis strains and building expensive temperature-controlled facilities that local actors considered unnecessary in the Jamaican tropical climate. One domestic cannabis entrepreneur described a deterioration of their relationship with an overseas partner following the investor’s pressure to change the distribution strategy:

About [year] they started to indicate that they weren’t interested in importing anything from Jamaica but they were more interested in, you know, satisfying the local market in Jamaica, which was not why we got into business [with them], I would not have done a deal with them had we known that export was not the primary goal. (KI14, industry).

Contracting legal expertise from the foreign investor’s home country was one strategy reported by local cannabis entrepreneurs to protect their own business interests, including for the scenario of dissolving a partnership. The potential for disagreements between local and international firms was also recognised by KIs from academia and the public sector, who expressed concern over the perceived imbalance of power between the two actors:

I am worried in a certain aspect of the level of influence that foreign investors will impose on the local industry. The local players have to be strong and they have to understand that there is something that you have that attracted the foreign investors to your space. (KI5, civil servant).

### Protecting Jamaica’s heritage and “landrace strains”

Many KIs from academia, civil society and the public sector expressed concern that foreign investors may threaten the genetic diversity of Jamaican sativa-dominant strains, which have adapted to growing in the island’s tropical climate. KIs observed that a significant part of the legal cultivation space in Jamaica is being dominated by foreign, more potent THC cannabis strains popular in North America (e.g. Girl Scout Cookies, OG Kush, Jack Herrer). There was also concern that investors focused on medical cannabis exports would pressure local firms to cultivate high-CBD cannabis varieties, resulting in the ‘squeezing out’ of heirloom strains from the market. In this context, local industry KIs also opposed the anticipated introduction of regulations for hemp (the draft is being considered as of mid-2020), due to the perceived threat of cross-pollination with ganja (cannabis) (e.g., KI9, 16, 19, industry), for example:

Hemp is a potential threat, it’s difficult … because the investors come in to plant hemp and they are not able to control that … they can wipe out our strains and that can put us into a big problem. (KI9, industry).

[Hemp farms] are the possible source of the contamination … that can possibly happen if you are not doing the right things. (…) In my opinion, and this is my humble opinion, for the space that we are as a country … it’s one versus the other, there’s going be a “hemporium” oil refinery-based country where, we’re growing hemp everywhere or we’re growing boutique cannabis and go with the [Jamaican] brand. (KI16, industry).

The latter quote illustrates local actors’ view that cannabis strains are part of Jamaica’s cultural heritage, and the need to “put measures in place to protect landraces” (e.g. K11, academic). While some domestic companies have entered into intellectual property agreements with foreign investors, this has proved challenging to some operators. One KI described how “a local licensee [was approached by] an international person who offered to buy his strain but he could never grow that strain again” (KI2, activist). While the details of this account are unverified, the quote illustrates KI’s concern over local actors’ ability to protect their interests. One KI went as far as to describe the threat as “piggy backing on brand Jamaica” (KI20, academic).

### The risks of political interference

Foreign investors’ economic power, while highly sought-after by domestic cannabis entrepreneurs, was also recognised as a potential risk factor for domestic political interference. For example, a couple of local cannabis entrepreneurs expressed the view that “the promise of prosperity” gives investors a lot of political bargaining power, particularly in the context of “underfunded government” in Jamaica (KI14, industry). KIs from the public sector and academia were also concerned over investors’ access to domestic policy elites and the pressure to develop and apply regulations in their favour. Two quotes below illustrate how they viewed the risk of corruption in this environment:

What they will do, some investors come into the country, they will indicate that they have a lot of money. They will need to apply [for a license] but they do not want to wait the time so they will also go to the Minister and complain and say that “the [CLA] staff is not doing …” but then they [Ministry] check the records and find the staff are following the rules. But sometimes they [investors] don’t really want to wait. (civil servant).

If I allow you to do certain things that are outside of the scope of regulation, it’s corruption. Corruption doesn’t necessarily mean that I have to take money from you, it means that I appear blind eye, I am seeing you breaking the law but I turn a blind eye - that’s also corruption. (KI15, academic).

Foreign investor’s bargaining power with political and government agency elites may benefit domestic cannabis businesses when the interests of domestic and foreign businesses are aligned, but may be a disadvantage when commercial interests diverge, as in the above case of facilitating the hemp sector. This adds to the complexity of relationships between the government, domestic cannabis businesses and overseas investors. One civil servant KI described how the domestic business community, rather than overseas investors, lobbied for lowering the 50% ‘majority ownership’ requirement for licensed cannabis operators, a change that in the long-term is likely to benefit foreign investors:

[There is a] constant pushback to tell the politicians to get rid of it, it has caused a lot of upset in the business community, so much so that you won’t have 51% for [future] hemp [regulations], it will be something far lower. Because there is significant pushback from the business community that, you know, they have to get money, to get into the research …, they can’t get 51% Jamaican ownership and you have to let a lot of foreigners, foreign money come in. (KI17, civil servant).

### Perceived impact on the inclusion of local farmers

Most KIs from academia, civil sector and public agencies viewed the opening of the domestic cannabis market to foreign investors as a potential opportunity to facilitate the transition of illegal cannabis farmers into the legal cannabis economy, primarily via financing of essential start-up infrastructure. However, the KIs recognised that achieving this objective depends on finding “responsible investors” with “commitment” to helping disadvantaged communities (e.g., KI20, academia).

When asked to reflect on the extent to which this has been achieved, KIs’ assessments ranged from “a work in progress” (KI20, academia) to a clear disappointment, with descriptions of local farmers being “side lined” by investors (e.g., KI7, civil servant) and KIs “worried about [local farmers] being squeezed out of the market” (K11, academic). One civil servant described how a highly anticipated partnership between an overseas company and a collective of local farmers “fell apart”, with the imbalance of economic power being the key reason:

Now that I’m thinking … you know, think of life … It is the ‘big pocket’ man who gets the show. So if you do something for the big pocket man, you are going to get the headlines, if you are doing something for the little man on the farm, you are not going to get the headlines... (KI17, civil servant).

In the context of the inherent vulnerability of illegal subsistence cannabis farmers, some KIs expressed the sentiment that the government should create a policy environment that supports the inclusion of small-scale growers into the legal market, thus arguing for a “more protectionist approach” (KI14, industry). KIs’ ideas included supporting a domestic cannabis farming sector via domestic government agricultural funding rather than foreign investment (e.g., KI21, politician, KI20, academic), and legal protections for domestic farmers, such as setting a minimum proportion of cannabis output that has to come from small-scale domestic farmers and setting a minimum purchase price paid by processors (KI12, civil servant):

There are many interests to be protected: our strains, ensure [our] farmers are entering to agreements that they can be comfortable with. (…) There’s a question: do you want to set a minimum price that the ganja [cannabis] should be sold [from collective farmers to wholesale buyers]. There should be some regional standards, there should be some regional acceptance that if investors come, they do so on a fair-trade basis. (KI12, civil servant).

## Discussion

This exploratory study investigated key cannabis policy stakeholders’ experiences with foreign investment in the emerging medicinal cannabis sector in Jamaica. Stakeholders from academia, and the public and private sectors all recognised the benefits of foreign capital, expertise and technology as a result of foreign commercial partnerships. Overwhelmingly, the focus of investments was on improving the efficiency of cannabis production and the linkages across the cannabis value chain from cultivation to processing. Some KIs expressed disappointment over limited commercial investment in the research and development activities that would add value to the domestic medicinal cannabis sector (see summary Table 1). This raises questions over the extent foreign investment will benefit the domestic actors in the long term. Research from other sectors, including the global pharmaceutical industry, has highlighted that directing investment into the creation of value-added products (i.e., original medical preparations) and protection of intellectual property via patents, copyrights and trademarks are key to ensuring a sustainable investment model, particularly in the context of developing economies [1].

**Table 1:** SWOT matrix of medicinal cannabis market and foreign investment strategy in Jamaica

**Strengths**	**Weaknesses**
- Jamaica’s cultural reputation - Genetic diversity of cannabis strains adapted to the local climate - Indigenous knowledge of cannabis cultivation	- Underfunded research and development (R&D) infrastructure - Lack of access to loans for domestic investors (due to banking arrangements with the US banks) - High cost of energy and security expenses
**Opportunities**	**Threats**
- Inflow of economic capital - Access to export markets - New legal employment sectors - Professionalisation of local sector via transfer of know-how and technology	- Risks of political interference - Social justice objectives of the reform (i.e., transitioning of illegal farmers to the legal sector) not prioritised - Pressure on local operators to adopt technologies considered unnecessary to Jamaican context - Investment focused only on the efficiency of production, lack of investment in research and development (R&D)

Ironically, the financial power of overseas investors - while highly sought after by domestic medicinal cannabis entrepreneurs – also created the risk of domestic political interference. While the interests of the domestic cannabis business sector often aligned with their foreign investor partners, as they share the common goal of generating revenue, KIs also described instances of diverging interests. One example of this was when an investor signalled a change in the planned distribution strategy (from export to the domestic market). International investors come with knowledge of sophisticated marketing strategies, which may have potential public health consequences for host countries should the market transition from medicinal to recreational. This health theme did not feature prominently in our KI interviews, perhaps reflecting Jamaica’s unique social context (with high availability of illegal cannabis and prevalence prior to the DDAA reform [62]) and the relative novelty of a legal retail market for therapeutic cannabis in Jamaica (with the first dispensary opened in March 2018).

Due to the time-lagged nature of implementation, the complexity of DDAA reform in Jamaica (i.e. 2015 reform also included decriminalisation of personal possession and allowance of home-growing), and the delays in measuring health indicators, it is too early to evaluate the (public) health impacts of the DDAA reform, such as changes in prevalence of cannabis use, patterns of cannabis use or substitution effects (with other pharmaceuticals as well as substances used recreationally such as alcohol). There are potentially positive and negative public health impacts of greater access to *medicinal* cannabis [23]. Interviews in this study illuminated mediating factors that could further help explain these effects (e.g. the extent of commercialisation and advertising vs. improved safety and quality of cannabis).

The issue of social justice and sustainable development of the new sector featured prominently in the interviews. Many KIs expressed concern that the entrance of international investors further focused policymakers’ attention on the economic aspects of the reform, pushing other considerations, including the social justice goal of transitioning illegal farmers to the legal sector further down the political agenda. This lack of priority could hinder the government action needed to reduce structural barriers faced by small scale illegal cannabis farmers (i.e., the costs of licensing and compliance with infrastructure and security requirements).

In mid-2017 the Jamaican government established a pilot Alternative Development Programme to facilitate communal cultivation of legal cannabis in two traditional illegal cannabis-growing communities, but the roll-out of this support has proved to be slow, with challenges in securing appropriate land title permissions [14, 37, 54]. This suggests that more government planning and regulation is needed in order to ensure the diversity of the sector. More recently, in June 2020 the CLA announced the development of a new (transitional) Special Permit license scheme [17]. The Scheme would be means-tested and allow traditional cannabis farmers to operate for 24 months without the need to comply with all infrastructural and security requirements [17]. The challenge of ensuring the inclusion of small-scale and community operators in new cannabis markets is not unique to Jamaica [2, 36]. The price declines caused by economies of scale gains in legal cannabis production regimes make it harder for small operators to compete with larger better-resourced firms [36].

This study has highlighted the complex dynamics between public officials, international investors, and two types of domestic cannabis industry players, i.e. licensed companies that come primarily from the Jamaican business sector (including returning diaspora) and small-scale farmers that rely on collective farming arrangements to enter the market. There are significant power imbalances between these two types of domestic cannabis industry actors, with the vulnerability of illegal cannabis farmers clearly recognised by KIs from the public and private sectors.

Other governments that have legalised medical and scientific cannabis regimes in recent years have adopted various policy approaches to protect domestic actors, including small-scale farmers, from foreign influence. For example, in Thailand, where medicinal cannabis production was legalised in 2019, the market is dominated by a state-owned agency, with only select categories of private actors allowed to participate (including agricultural cooperatives and community enterprises) [6]. Foreign investment and imports will be prohibited for five years to allow the domestic medical cannabis sector to become established [55]. In St Vincent and Grenadines, a Caribbean island that legalised cannabis for medicinal and scientific purposes in 2018, traditional illegal growers of cannabis are encouraged to transition into the legal market via a special legislated amnesty process, and new investors need to purchase at least 10% of all cannabis raw material for processing from these traditional farmers [30, 49]. A similar legal requirement that 10% of production should come from small and medium scale growers was included in the law reform in Colombia, but the impact on diversity of the sector has been described as modest at best [47]. Evaluation of these different approaches is needed to determine best practice policy to protect the diversity of domestic markets and host country interests.

The objective of boosting local economies via cannabis exports and foreign investments has been an important motivation for the legalisation of medicinal cannabis in Jamaica, and indeed in other countries with developing economies. However, the demand for cannabis produced in those countries remains debatable. Following the completion of interviews for this study, some international partners of Jamaican cannabis businesses announced the withdrawal of their investments from the Caribbean [57]. Reportedly, the announcement was made in the context of cannabis companies downsizing operations (partly due to fallout from the coronavirus pandemic) and limited opportunities for international cannabis exports. While some media sources suggested it was also linked to the slow progress with finalising official regulations for commercial exports by Jamaican authorities [57], the claim has been strongly refuted by public officials [16]. This recent development illustrates the fragility of business partnerships between local cannabis firms and foreign investors.

## Conclusions

While foreign investment has facilitated the commercialisation and professionalisation of the medicinal cannabis sector in Jamaica, regulatory measures are also needed to protect the domestic industry and support the transition of small-scale illegal cultivators to the legal regime. Due to the time-lagged nature of implementation, the complexity of DDAA reform, and the delays in measuring health indicators, it is too early to evaluate the public health impacts of the reforms. The study has discussed how economic, social and political determinants of health may be altered by the presence of foreign investors in the medicinal cannabis sector.

### Limitations

The study explored experiences with foreign investors in an emerging cannabis market, drawing on observations of information-rich key informants in Jamaica. KIs’ views on foreign investment reflected both their role in the regime and their personal views on balancing the economic and social justice goals of the reform. We interviewed a range of actors from different sectors in an effort to provide a balanced analysis reflecting the diversity of perspectives and experiences of key cannabis policy stakeholders in Jamaica. The research explored issues from a host country perspective, therefore foreign investors were not interviewed. The official government data sources on the extent of foreign investment in cannabis market in Jamaica are limited. The KI interview method allowed access to information otherwise unavailable in the public domain, while providing relevant social, cultural and policy context.

## Data Availability

The data that supports the findings is available in the article.The larger dataset generated and analysed during the current study are not publicly available due to confidentiality reasons (i.e. contains information that could compromise research participant privacy/consent).

## Footnotes

1 Although the DDAA legislation does not restrict product types that can be sold, the Ministry of Health current position is not to allow products in edible form [15, 50]
